# Control of proline accumulation under drought via a novel pathway comprising the histone methylase CAU1 and the transcription factor ANAC055

**DOI:** 10.1093/jxb/erx419

**Published:** 2017-12-14

**Authors:** Yanlei Fu, Hailing Ma, Siying Chen, Tianyu Gu, Jiming Gong

**Affiliations:** 1 National Key Laboratory of Plant Molecular Genetics, CAS Center for Excellence in Molecular Plant Sciences, Institute of Plant Physiology and Ecology, Shanghai Institutes for Biological Sciences, Chinese Academy of Sciences, Shanghai, People’s Republic of China; 2 University of Chinese Academy of Sciences, Beijing, People’s Republic of China

**Keywords:** *ANAC055*, CAU1, drought tolerance, histone methylase, *P5CS1*, proline

## Abstract

Proline plays a crucial role in the drought stress response in plants. However, there are still gaps in our knowledge about the molecular mechanisms that regulate proline metabolism under drought stress. Here, we report that the histone methylase encoded by *CAU1*, which is genetically upstream of *P5CS1* (encoding the proline biosynthetic enzyme Δ^1^-pyrroline-5-carboxylate synthetase 1), plays a crucial role in proline-mediated drought tolerance. We determined that the transcript level of *CAU1* decreased while that of *ANAC055* (encoding a transcription factor) increased in wild-type Arabidopsis under drought stress. Further analyses showed that CAU1 bound to the promoter of *ANAC055* and suppressed its expression via H4R3sme2-type histone methylation in the promoter region. Thus, under drought stress, a decreased level of CAU1 led to an increased transcript level of *ANAC055*, which induced the expression of *P5CS1* and increased proline level independently of *CAS*. Drought tolerance and the level of proline were found to be decreased in the *cau1 anac055* double-mutant, while proline supplementation restored drought sensitivity in the *anac055* mutant. Our results reveal the details of a novel pathway leading to drought tolerance mediated by *CAU1*.

## Teaser

Expression of *CAU1* (encoding a histone methylase) decreases under drought, leading to de-repression of the *NAC* transcription factor *ANAC055* and its genetically downstream proline synthetase gene *P5CS1*, and this results in proline accumulation and a consequent increase in drought tolerance.

## Introduction

In plants under osmotic stress, proline is mainly synthesized by P5CS (Δ^1^-pyrroline-5-carboxylate synthetase) (Savouré *et al.*, 1995; Yoshiba *et al.*, 1995; Székely *et al.*, 2008) and P5CR (P5C reductase) from glutamate in chloroplasts (Szoke *et al.*, 1992; Verbruggen *et al.*, 1993). Proline catabolism is controlled by PDH (proline dehydrogenase) (Kiyosue *et al.*, 1996; Verbruggen *et al.*, 1996) and P5CDH (P5C dehydrogenase) in Arabidopsis (Deuschle *et al.*, 2001). As well as functioning as a compatible osmolyte, proline may act as a metabolic signal that regulates the stabilization of proteins and antioxidant enzymes, the direct scavenging of ROS (reactive oxygen species), and the balance of intracellular redox homeostasis, such as the ratios of NADP^+^/NADPH and GSH/GSSG (Hamilton and Heckathorn, 2001; Kavi Kishor *et al.*, 2005; Miller *et al.*, 2009; Szabados and Savouré, 2010; Alves *et al.*, 2011).

Several studies have shown that the transcription of *P5CS1* is activated by H_2_O_2_-derived signals, the calcium signal, PLC (phospholipase C), PLD (phospholipase D), and by the ABA-dependent pathway (Yoshiba *et al.*, 1995; Savouré *et al.*, 1997; Strizhov *et al.*, 1997; Parre *et al.*, 2007; Verslues *et al.*, 2007; Ghars *et al.*, 2008). *ABI1* (*ABA-INSENSITIVE 1*) and the CaM4 calmodulin-MYB2 regulatory pathway are involved in the control of *P5CS1* transcription (Knight *et al.*, 1997; Strizhov *et al.*, 1997; Yoo *et al.*, 2005; Parre *et al.*, 2007). Although much is known about the biological functions of proline in stress tolerance, its regulation needs further investigation.

In plants, histone modifications have been implicated in the response to drought stress (Sokol *et al.*, 2007; van Dijk *et al.*, 2010; Kim *et al.*, 2012; Sani *et al.*, 2013; Zong *et al.*, 2013). The dehydration-stress response gene *ATX1* encodes a protein that trimethylates histone H3 at lysine 4 (H3K4me3) in *NCED3* (*NINE-CIS-EPOXYCAROTENOID DIOXYGENASE 3*) (Ding *et al.*, 2009, 2011). The H3K4 demethylase homolog HvPKDM7-1 may be involved in drought tolerance in barley (Papaefthimiou and Tsaftaris, 2012). The histone acetylation levels increase in *RD20* (*RESPONSIVE TO DEHYDRATION 20*), *RD29A* (*RESPONSIVE TO DESICCATION 29A*), and *RD29B* (*RESPONSIVE TO DESICCATION 29B*) in Arabidopsis, and in four HATs (Histone acetyltransferases) genes *OsHAC703*, *OsHAG703*, *OsHAF701*, and *OsHAM701* in rice under drought stress (Kim *et al.*, 2008; Fang *et al.*, 2014). Histone deacetylase 2, encoded by *AtHD2C*, is involved in tolerance to drought stress in Arabidopsis (Sridha and Wu, 2006). Histone H4 deacetylation is also involved in ABA-induced stomatal closing (Sridha and Wu, 2006; Zhu *et al.*, 2008). However, there is still much to learn about the mechanisms of histone modification under drought stress.

Previously, we showed that the H4R3sme2-type histone methylase *CAU1/PRMT5/SKB1* mediates stomatal closure by repressing the expression of *CAS* (*CALCIUM SENSOR*, mediating the sensing of extracellular Ca^2+^ in guard cells) in response to extracellular calcium (Han *et al.*, 2003; Fu *et al.*, 2013). However, the *cas-1* mutant showed a partly restored water-loss rate and the same rate of stomatal closure as that of the wild-type (Fu *et al.*, 2013), suggesting that other components may function in the drought tolerance pathway mediated by *CAU1*.

In this study, we show that the transcription factor encoded by *ANAC055* acts as a downstream component of *CAU1* independently of *CAS*. Our results show that drought stress represses the levels of *CAU1* RNA and CAU1 protein, which lead to decreased H4R3sme2 methylation levels of chromatin in the *ANAC055* promoter. The subsequent increase in *ANAC055* expression leads to increased expression of its genetically downstream gene, *P5CS1*, resulting in proline accumulation and drought tolerance.

## Materials and methods

### Plant material, growth conditions, and physiological analyses

Plants of *Arabidopsis thaliana* were grown in soil at 22 °C with 16-h light/8-h dark cycles. At 14 d after emergence, drought stress was induced by withholding watering for 14 d, and the survival rate was scored at 7 d after watering recommenced. Rosette leaves of 22-d-old sample plants were collected and used to determine rates of water loss by time-course measurements of their fresh weights (Vartanian *et al.*, 1994; Pei *et al.*, 1998). Stomatal assays were performed as previously described (Pei *et al.*, 2000; Fu *et al.*, 2013). Stomatal apertures were determined by measuring the pore widths and lengths with a digital ruler in Image-Pro Plus 6.0 (MediaCybernetics).

Alternatively, plants were grown in quarter-strength hydroponics as previously described (Arteca and Arteca, 2000; Gong *et al.*, 2003). At 4 weeks of age, plants were treated with 10%, 20%, 30%, or 40% PEG-6000 and/or 50 mM proline for 12 h, or with 10% PEG-6000 for 0, 1, 3, 6, 12 h. Shoots were sampled and subjected to further analyses as indicated. Plants were weighed at the start of treatment (initial weight) and then reweighed at the end of treatment (final weight). Plants were then dried to a constant weight at 80 °C and reweighed to obtain dry weight. RWC was calculated as: (final weight – dry weight)/(initial weight – dry weight) ×100%.

### DNA constructs and plant transformation

The *ANAC055* cDNA was amplified by RT-PCR. The two restriction sites for *Bam*HI and *Sac*I were introduced using *ANAC055-1* primers and for *Xho*I and *EcoR*I using *CAU1*-1 (see Table S1 available at the Dryad Digital Repository, https://doi.org/10.5061/dryad.hc4bj). The resulting fragments were confirmed by sequencing and then sub-cloned into the binary vector pBI121 (pre-digested with *Bam*HI and *Sac*I) or *35S:EYFP*/pMON530 (pre-digested with *Xho*I and *EcoR*I). The *ANAC055* promoter was amplified by RT-PCR. The two restriction sites for *Pst*I and *Bam*HI were introduced using *ANAC055-2* primers (see Table S1 at Dryad). The resulting fragments were confirmed by sequencing and then sub-cloned into the binary vector *GUS*/pCAMBIA1300 (pre-digested with *Pst*I and *Bam*HI). The generated constructs *35S:ANAC055*/pBI121, *35S:EYFP-CAU1*/pMON530 and *pANAC055:GUS/*pCAMBIA1300 were transformed into Col-0 or *cau1* using the floral dip method (Clough and Bent, 1998). Transgenic lines with a segregation rate of 3:1 grown on kanamycin or hygromycin plates were used for further homozygote and strong allele screenings.

### RT-PCR/quantitative RT-PCR

Total RNA from plants was isolated using the TRIzol reagent (Invitrogen). First-strand cDNA synthesis, RT-PCR, and quantitative RT-PCR (qPCR) were performed as previously described (Aggarwal *et al.*, 2010). *ANAC055-QP*, *CAU1-QP*, *P5CS1-QP*, *SAND-QP*, and drought-related gene primers were used in quantitative RT-PCR (see Table S1 at Dryad).

### Histochemical analysis

Transgenic plants of the *pANAC055*:*GUS*/*cau1* mutant were subjected to histochemical analysis as previously described (Aggarwal *et al.*, 2010).

### Isolation of *anac055* and *cau1 anac055* mutant plants

The T-DNA insertion line SALK_014331 obtained from the Arabidopsis Biological Resource Center (https://abrc.osu.edu/) was screened for the homozygous knockout mutant *anac055* as previously described (Weinl *et al.*, 2008). To generate the *cau1 anac055* double-mutant, *cau1* was crossed to *anac055* to make an F_2_ population; *cau1*-like plants were further analysed to isolate the genotype *anac055/anac055* using the PCR primers *ANAC055*-SALK and LBA1 as previously described (Krysan *et al.*, 1999).

### Determination of proline levels

Proline concentrations were determined as described by Bates *et al.* (1973). Leaves were freeze-dried and then homogenized in 3% sulfosalicylic acid, and were then centrifuged at 3000 *g* for 20 min. The sample supernatant, acetic acid, and 2.5% acid ninhydrin solution were boiled for 30 min, and the absorbance was measured at 520 nm.

### Chromatin immunoprecipitation (ChIP) assay

For the ChIP assay, 21-d-old Col-0, *cau1*, and *35S*:*EYFP*-*CAU1*/*cau1* plants grown under long-day conditions were harvested. Approximately 4 g of plant material was cross-linked for 20 min in 1% formaldehyde. ChIP assays were performed as previously described (Vartanian *et al.*, 1994; Ascenzi and Gantt, 1999). The sonicated chromatin extractions were immunoprecipitated overnight with antisymmetric dimethyl-H4R3 antibody (Abcam) for plants of Col-0 and *cau1*, with an anti-GFP (green fluorescent protein) antibody (Invitrogen) for plants of *35S*:*EYFP*-*CAU1*/*cau1*, or without antibody. Incubation of chromatin with mouse IgG (Abcam) served as a mock immunoprecipitation control. Protein A beads (Millipore) were used to capture the immunocomplexes. After reverse cross-linking and proteinase-K digestion, the DNA was extracted with phenol-chloroform and then precipitated with ethanol. The immunoprecipitated DNA was subsequently used for qPCR. The sequences were amplified from –1388 to 646 bp of the *ANAC055* gene and each DNA fragment was approximately 120 bp in length. Primers used for ChIP-qPCR were as follows: Region A (*ANAC055-1*), region B (*ANAC055-2*), region C (*ANAC055-3*), region D (*ANAC055-8*), region E (*ANAC055-9*), region F (*ANAC055-10*), region G (*ANAC055-11*), and the primer sequences are given in Table S1 at Dryad. *TUB8* was used as a control (Mathieu *et al.*, 2005).

### Protein gel blotting analysis

Transgenic *35S*:*EYFP*-*CAU1*/*cau1* plants were grown in hydroponics to 24 days of age, and then exposed to 10% PEG treatments. Total proteins were extracted from leaf samples using buffer E [125 mMTris-HCl pH 8.0; 1% (w/v) SDS, 10% (v/v) glycerol, 50mM NaS_2_O_5_]. From each sample 30 µg total proteins were separated on 12% SDS-PAGE (Beyotime) gel and analysed by protein gel blotting according to the manufacturer’s instructions. Mouse anti-Actin2 and anti-GFP (Abmart; at 1:5000 dilution) were used as primary antibodies. The membranes were visualized using a Super-Signal West Pico Chemiluminescent Substrate Kit (Thermo Scientific) according to the manufacturer’s instructions.

### Statistical analysis

Data were statistically analysed using one-way ANOVA with LSD tests (for multiple comparisons) or two-tailed Student’s *t*-tests (for comparisons of two sets of data).

## Results

### *ANAC055* expression is enhanced in the *cau1* mutant

**Fig. 1.**
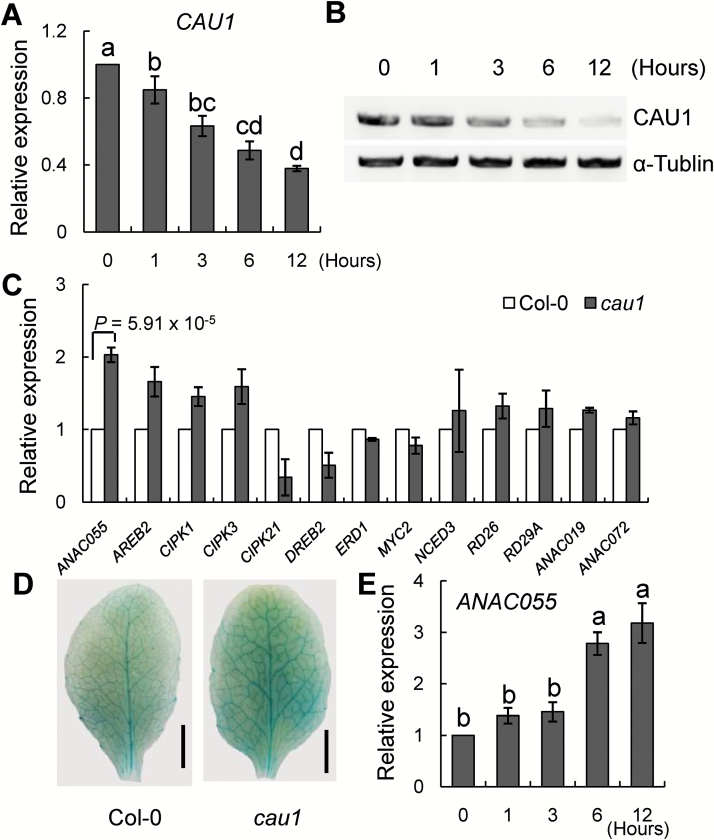
Enhanced expression of *ANAC055* in the *cau1* mutant compared with Col-0. (A) Decrease in *CAU1* transcript levels in Col-0 under drought stress. Transcript levels of *CAU1* in 4-week-old hydroponically grown plants treated with 10% PEG-6000 for 0, 1, 3, 6, and 12 h. *SAND* (AT2G28390) was used as an internal control. Values are means ±SE, *n*=3. (B) Decrease in CAU1 protein levels in Col-0 under drought stress. CAU1 detected by immunoblotting in 4-week-old plants exposed to 10% PEG-6000 treatment for 0, 1, 3, 6, and 12 h. (C) Relative transcript levels of genes functioning in the drought tolerance pathway as determined by RT-PCR. For each gene, the transcript level was set to 1 in Col-0. (D) Expression of *ANAC055* promoter–GUS fusions in leaves of Col-0 and the *cau1* mutant. Scale bars =0.2 cm. (E) Increase in *ANAC055* transcript levels in Col-0 under drought stress. Transcript levels of *ANAC055* in 4-week-old plants treated with 10% PEG-6000 for 0, 1, 3, 6, and 12 h. (This figure is available in colour at *JXB* online.)

Previously, we showed that the *cau1* mutant is resistant to drought stress (Fu *et al.*, 2013). To investigate the role of *CAU1* in drought tolerance, the levels of *CAU1* transcripts and CAU1 protein were determined in the wild-type (Col-0) and the *cau1* mutant under drought stress conditions. The *CAU1* transcript levels (Fig. 1A) and CAU1 protein levels (Fig. 1B) in Col-0 decreased under drought stress. Next, we analysed the transcript levels of drought tolerance-related genes including *ANAC055*, *AREB2*, *CIPK1*, *CIPK3*, *CIPK21*, *DREB2*, *ERD1*, *MYC2*, *NCED3*, *RD26*, *RD29A, ANAC019*, and *ANAC072* by quantitative RT-PCR. The transcript level of *ANAC055*, which encodes a transcription factor, was enhanced in the *cau1*mutant (Fig. 1C), compared with that in Col-0. Histochemical analyses showed that the activity of GUS driven by the *ANAC055* promoter was higher in *cau1* leaves than in Col-0 leaves (Fig. 1D). The transcript level of *ANAC055* in Col-0 was enhanced under drought stress (Fig. 1E). These results indicated that the *ANAC055* level was enhanced in *cau1*, and that drought stress suppressed *CAU1* expression but increased *ANAC055* expression.

### CAU1 regulates *ANAC055* expression by H4R3sme2 methylation in its promoter region

**Fig. 2.**
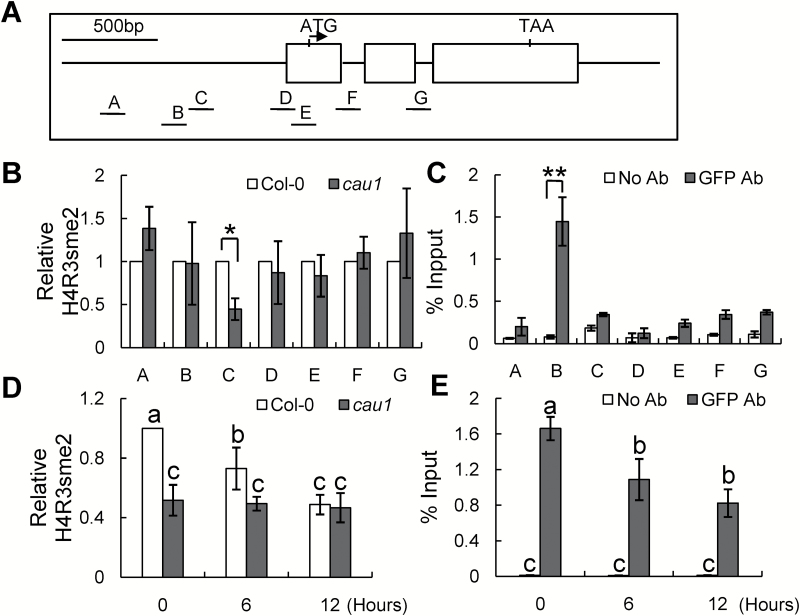
Suppression of CAU1-mediated histone methylation in the *ANAC055* promoter region under drought stress. (A) Diagram of the *ANAC055* gene. The genomic regions A to G used in the ChIP assays are indicated. (B, C) ChIP assays with antibodies against H4R3sme2 (B) using Col-0 and *cau1* plants, and GFP (C) using *35S*:*EYFP*-*CAU1*/*cau1* plants. *TUB8* was used as an internal control in (C). Three independent experiments were performed, and values are means ±SE. *P<0.05 and **P<0.01. (D, E) ChIP assays with antibodies against H4R3sme2 within the C region (D) or GFP within the B region (E) of *ANAC055*. Plants were treated with 10% PEG-6000 for the period indicated. Three independent experiments were performed. *TUB8* was used as an internal control in (D). Values are means ±SE. Different letters above each bar indicate significant differences (ANOVA tests).

To explore the mechanism by which CAU1 regulates *ANAC055* expression, we performed a ChIP-qPCR assay to analyse the H4R3sme2 level in the *ANAC055* promoter region, using an H4R3sme2 antibody (Fig. 2A, B). The H4R3me2 level in region C of the *ANAC055* promoter was significantly reduced in *cau1* (Fig. 2A, B). This result suggested that CAU1 regulated *ANAC055* transcription through histone methylation.

We also conducted a ChIP-qPCR assay using a GFP antibody to determine whether CAU1 binds to the *ANAC055* chromatin (Fig. 2A, C). CAU1 strongly associated with region B of the *ANAC055* promoter, whereas a similar CAU1–*ANAC055* interaction was not detected in regions A or C–G. These results confirmed that CAU1 bound directly to the *ANAC055* chromatin in region B, and mediated the level of histone methylation in region C (Fig. 2A–C).

Given that *ANAC055* expression was up-regulated in response to drought stress (Fig. 1E), we analysed the correlation between drought stress and the level of H4R3sme2 in the *ANAC055* promoter. As shown in Fig. 2D, drought stress significantly decreased the H4R3sme2 level in region C of the *ANAC055* promoter in Col-0, while no change was observed in the *cau1* mutant. Further analyses showed that there were significant decreases in CAU1 binding to the *ANAC055* promoter (Fig. 2E) as well as significant decreases in *CAU1* mRNA and CAU1 protein levels in Col-0 under drought stress (Fig. 1A, B). These data indicated that drought stress decreased the *CAU1* mRNA and CAU1 protein levels and decreased CAU1 binding to the *ANAC055* promoter, thus decreasing H4R3sme2 methylation of the *ANAC055* chromatin and enhancing *ANAC055* expression.

### *CAU1* acts with *ANAC055* in response to drought stress

A previous study showed that the *NAC* gene family member *ANAC055* was up-regulated by drought stress, and its over-expression increased drought tolerance (Tran *et al.*, 2004). Given that *cau1* was shown to be insensitive to drought stress (Fu *et al.*, 2013) and showed enhanced *ANAC055* expression (Fig. 1C), we sought to determine whether these phenotypes were genetically correlated with *ANAC055*. A T-DNA insertion line for *ANAC055* was isolated (see Fig. S1A, B at Dryad). RT-PCR analysis confirmed that *ANAC055* mRNA was not detectable in the *anac055* mutant (see Fig. S1C at Dryad).

**Fig. 3.**
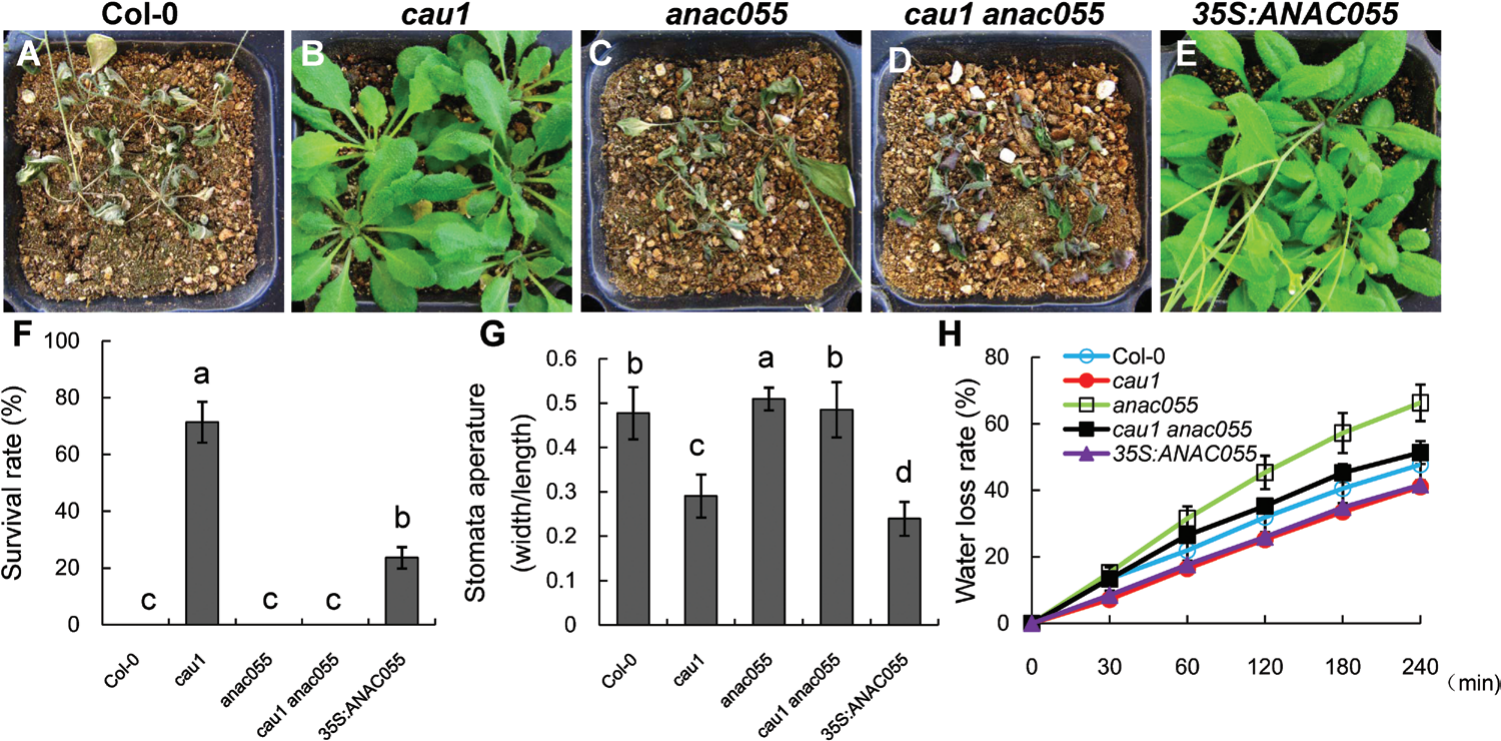
*CAU1*–*ANAC055* acts in the drought tolerance pathway. (A–E) Drought tolerance of Col-0 (A), *cau1* (B), *anac055* (C), *cau1 anac055* (D), and *35S:ANAC055* (E). When plants were 14 d old watering was withheld for 14 d, and then watering recommenced for 7 d. (F) Survival rates of plants in (A–E) under drought stress. Values are means ±SD from three independent experiments. (G) Stomatal apertures on rosette leaves. Values are means ±SD (*n*≥30). (H) Water loss rates from leaves of Col-0, *cau1*, *anac055*, *cau1 anac055*, and *35S:ANAC055.* Values are means ±SD from three independent experiments (*n*=6 leaves per treatment). Different letters above each bar indicate significant differences (ANOVA tests).

Further analyses showed that Col-0 (Fig. 3A, F) and *anac055*, the loss-of-function mutant of *ANAC055* (Fig. 3C, F), were sensitive to drought stress, while *cau1* plants were drought tolerant (Fig. 3B, F). However, the drought tolerance conferred by the *cau1* mutation was abolished in the double mutant *cau1 anac055* (Fig. 3D, F), even though the visible developmental phenotypes of *cau1 anac055* were similar to those of *cau1*. A drought-tolerant phenotype was also observed in *35S:ANAC055* (Fig. 3E, F). These results indicated that *ANAC055* is downstream of *CAU1*, which functions in drought tolerance.

Next, we evaluated differences in stomatal apertures among the mutants and Col-0. As shown in Fig. 3G, the stomatal aperture was smaller in *cau1* than in Col-0, while that of *anac055* was larger than that of Col-0. In the double-mutant *cau1 anac055*, stomatal aperture was restored to a level between those of Col-0 and *anac055* (Fig. 3G). The rate of water loss from the leaves was decreased in *cau1* and *35S:ANAC055*, and increased in *anac055* compared with that of Col-0 (Fig. 3H). These results suggested that *ANAC055* functions downstream of *CAU1,* and plays an important role in stomatal closure and consequently in drought tolerance.

### *CAU1* affects proline accumulation via its effects on *P5CS1*

**Fig. 4.**
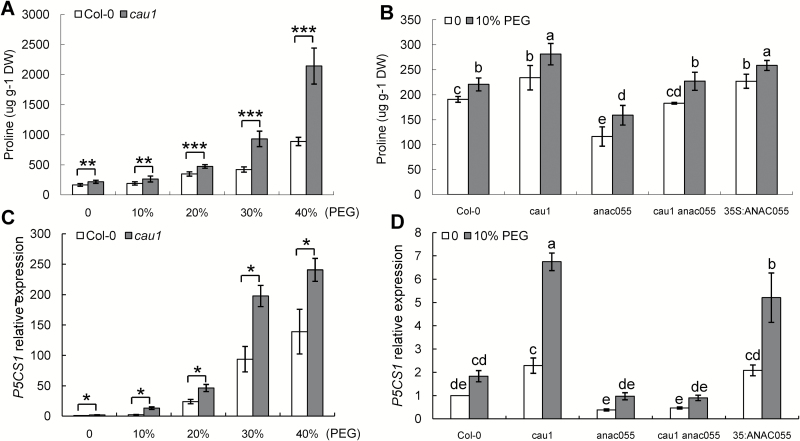
*CAU1* regulates proline metabolism via *P5CS1*. (A, B) Proline levels in shoots. *n*=6 in (A), 3–6 in (B). (C, D) Transcript levels of *P5CS1* in shoots as determined by RT-PCR. *SAND* (AT2G28390) was used as an internal control. Values are means ±SE, *n*=3. *P<0.05, **P<0.01, and ***P<0.001. Different letters above each bar indicate significant differences (ANOVA tests).

To elucidate the molecular mechanism of *CAU1* in the drought response, we measured the proline levels in Col-0 and *cau1* under drought stress imposed by PEG-6000. Proline accumulated in both Col-0 and *cau1* under drought stress with a clear dose-dependent effect, and to higher levels in *cau1* than in Col-0 (Fig. 4A). In the absence of drought stress, there were higher proline contents in *cau1*and *35S:ANAC055* than in Col-0 and *cau1 anac055*, but lower proline content in *anac055* than in Col-0 (Fig. 4B). The transcript levels of *P5CS1* in Col-0 and *cau1* also increased under drought stress with a dose-dependent effect, and to higher levels in *cau1* than in Col-0 (Fig. 4C). In the absence of drought stress, the *P5CS1* transcript levels were higher in *cau1* and *35S:ANAC055* than in Col-0, but lower in *anac055* and *cau1 anac055* than in Col-0 (Fig. 4D). The proline level and *P5CS1* transcript levels increased in plants in response to PEG-6000. Higher proline contents and *P5CS1* transcript levels existed in *cau1* and *35S:ANAC055* than in Col-0 and *cau1 anac055*, and lower proline content and *P5CS1* transcript levels existed in *anac055* than in Col-0 (Fig. 4B, D).

**Fig. 5.**
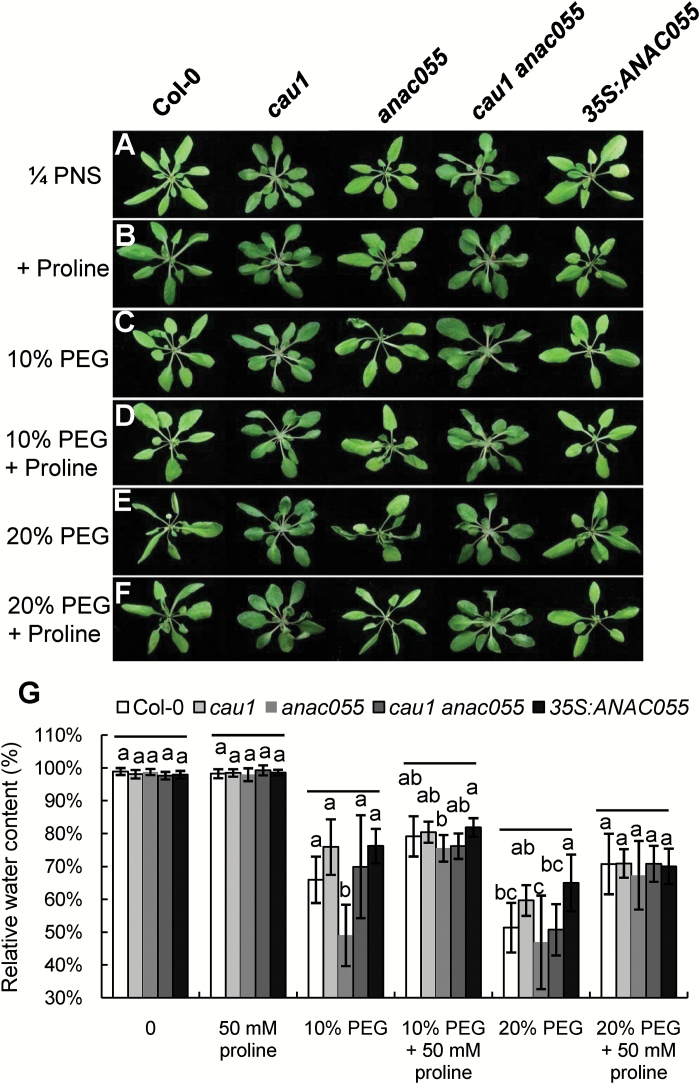
Phenotypic analysis of plants exposed to drought stress (PEG-6000) and proline. Hydroponically grown 4-week-old plants of Col-0, *cau1*, *anac055*, *cau1 anac055,* and *35S:ANAC055* are shown. (A) Control plants grown in 1/4 plant nutrient solution (PNS). (B) Plants treated with 50 mM proline for 12 h. (C) Plants treated with 10% PEG-6000 for 12 h. (D) Plants treated with 10% PEG-6000 and 50 mM proline for 12 h. (E) Plants treated with 20% PEG-6000 for 12 h. (F) Plants treated with 20% PEG-6000 and 50 mM proline for 12 h. (G) Relative water content from plants of Col-0, *cau1*, *anac055*, *cau1 anac055*, and *35S:ANAC055.* Values are means ±SD from three independent experiments (*n*=6–7 per treatment). Different letters above each bar indicate significant differences (ANOVA tests) among the five samples in one treatment.

 To identify the role of *CAU1*–*ANAC055* in drought tolerance via the regulation of *P5CS1*, we tested whether proline could restore the sensitivity to osmotic stress imposed by PEG-6000 in *anac055* plants. When treated with 10% (Fig. 5C, G) or 20% PEG (Fig. 5E, G) for 12 h, Col-0 and *cau1 anac055* showed wilting symptoms and decreased RWC in a dose-dependent manner compared with their respective untreated controls (Fig. 5A, G) or with proline-treated plants (Fig. 5B, G). *cau1* and *35S:ANAC055* plants showed higher RWC under 10% or 20% PEG treatment compared with those of Col-0 and *cau1 anac055* (Fig. 5C, E, G). *anac055* plants showed lower RWC under 10% or 20% PEG treatment compared with those of Col-0 and *cau1 anac055* (Fig. 5C, E, G).

Proline restored the RWC of Col-0, *cau1*, *anac055*, *cau1 anac055*, and *35S:ANAC055* plants treated with 10% or 20% PEG (Fig. 5G). Plants showed mild wilting in a dose-dependent manner when treated with 10% PEG and proline and 20% PEG and proline (Fig. 5D, F, G) compared to those treated with 10% and 20% PEG without proline (Fig. 5C, E, G). Under the same treatments, *cau1 anac055* showed similar phenotypes to those of Col-0, and *35S:ANAC055* showed similar phenotypes to those of *cau1* (Fig. 5). These results indicated that *CAU1–ANAC055* acts in drought tolerance by regulating the expression of *P5CS1* and consequently the proline level.

### The *CAU1–ANAC055* pathway acts independently of the *CAU1–CAS* pathway in drought tolerance

**Fig. 6.**
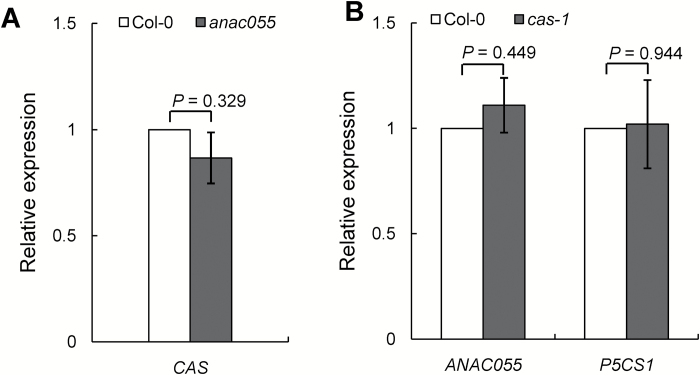
Analyses of transcript levels of *CAS* in the *anac055* mutant, and *ANAC055* and *P5CS1* in the *cas-1* mutant. (A) RT-PCR amplification of *CAS* in *anac055*. (B) RT-PCR amplification of *ANAC055* and *P5CS1* in *cas-1*. *SAND* (AT2G28390) was used as an internal control. Values are means ±SE, *n*=3. Statistical significance was determined by *t*-tests.

**Fig. 7.**
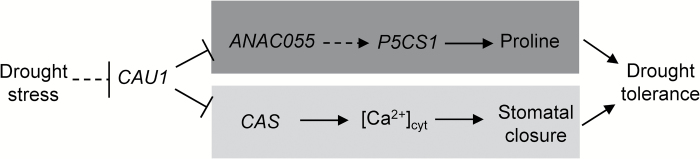
A model of drought tolerance mediated by *CAU1*–*ANAC055* and proline. Drought stress reduces *CAU1* mRNA and CAU1 protein levels, and decreases the binding of CAU1 to the promoter of *ANAC055* (dark grey box). This decreases histone methylation of the *ANAC055* chromatin, leading to increased *ANAC055* expression. The subsequent increase in *P5CS1* expression leads to increased proline levels and enhanced drought tolerance. The *CAU1*–*ANAC055* pathway (dark grey box) may play a redundant role with the *CAU1*–*CAS* pathway (light grey box) to mediate plant adaptation to drought. Dashed lines represent possible unidentified steps, or steps that have been identified but are not shown here.

Previously, we showed that the *CAU1*–*CAS* pathway regulates stomatal closure (Fu *et al.*, 2013). To investigate whether *ANAC055* is involved in the *CAU1*–*CAS* pathway, we analysed the transcript levels of *CAS* in the *anac055* mutant and the transcript levels of *ANAC055* and *P5CS1* in the *cas-1* mutant. The transcript levels of *CAS* in the *anac055* mutant (Fig. 6A) and of *ANAC055* and *P5CS1* in the *cas-1* mutant (Fig. 6B) were similar to those in Col-0. These results suggested that *CAU* regulates *ANAC055* independently of *CAS* in drought tolerance (Fig. 7).

## Discussion

Proline acts as an osmolyte and a signaling molecule to modulate responses to abiotic and biotic stresses (Szabados and Savouré, 2010), but the regulation of proline synthesis is not completely understood. In this study, CAU1, an H4R3sme2-type histone methylase, was shown to bind to the promoter region of *ANAC055* and repress its expression. The decrease in CAU1 under drought stress led to higher expressions of *ANAC055* and its genetically downstream gene *P5CS1*.

### CAU1 regulates expressions of *ANAC055* and *P5CS1* in response to drought stress

In plants, proline accumulates under abiotic stress (Saradhi *et al.*, 1995; Yoshiba *et al.*, 1995; Schat *et al.*, 1997; Choudhary *et al.*, 2005; Yang *et al.*, 2009) and biotic stress (Fabro *et al.*, 2004; Haudecoeur *et al.*, 2009). Proline synthetase (P5CS1) is regulated by calcium signals, H_2_O_2_-derived signals, and an ABA-dependent pathway (Yoshiba *et al.*, 1995; Savouré *et al.*, 1997; Strizhov *et al.*, 1997; Parre *et al.*, 2007; Verslues *et al.*, 2007). The known regulators in plants include CaM4-MYB2 (Yoo *et al.*, 2005), PLC (Knight *et al.*, 1997; Parre *et al.*, 2007), PLD (Thiery *et al.*, 2004; Ghars *et al.*, 2008), ABI (Strizhov *et al.*, 1997), LOS5/ABA3 (Xiong *et al.*, 2001), LcMYB1 (Cheng *et al.*, 2013), SpERD15 (Ziaf *et al.*, 2011), GmbZIP132 (Liao *et al.*, 2008), GsZFP1 (Luo *et al.*, 2012), and TaABC1 (Wang *et al.*, 2011).

Our results show that *CAU1* represses the expression of *ANAC055*; thus, the decrease in CAU1 under drought stress leads to higher transcript levels of *ANAC055* and the genetically downstream *P5CS1*, resulting in increased proline synthesis. However, the complete NACRS (NAC recognition sequence, TCNNNNNNNACACGCATGT) was not determined in the *P5CS1* region (Tran *et al.*, 2004). *P5CS1* might act genetically downstream of *ANAC055* while not being directly bound in the upstream region with the ANAC055 protein.

*CAU1*–*ANAC055* affects the gene expression involved in proline metabolism. The expression of *P5CR*, involved in the biosynthesis of proline, was higher in *cau1* and *35S:ANAC055* than in Col-0 and *cau1 anac055*, and lower in *anac055* than in Col-0 (see Fig. S2 at Dryad). In terms of the higher level of *P5CS1*, GSA/P5C might be the major factor that enhanced the level of *P5CR* in *cau1* and *35S:ANAC055*. The expression of *PDH1* and *P5CDH*, involved in the catabolism of proline, tended to be lower in *cau1* and *35S:ANAC055* than in Col-0 and *cau1 anac055*, and higher in *anac055* than in Col-0 (see Fig. S2 at Dryad). These observations might be the result of changed levels of proline in the mutants. The results support a model wherein CAU1 functions upstream of a P5CS1–proline cascade (Fig. 7).

Proline functions as an osmolyte and also as a signaling molecule to regulate metabolite pools and the redox balance, to control gene expression, and ultimately to control drought tolerance (Szabados and Savouré, 2010). The transcript levels of *P5CS1* were enhanced in the *cau1* mutant and suppressed in the *anac055* and *cau1 anac055* mutants (Fig. 4D). The proline level in *cau1 anac055* was partly restored to a level similar to that in Col-0, but the level in the *anac055* mutant was very low (Fig. 4B). These results indicate that there must be other components besides *P5CS1* that function in *CAU1*-mediated proline synthesis.

A previous study showed that the transcript levels of three *NAC* transcription factors, *ANAC055*, *ANAC019*, and *ANAC072*, were increased under drought stress, and transgenic plants overexpressing these factors showed increased drought tolerance (Tran *et al.*, 2004). In the present study, however, the transcript level of *ANAC055* was up-regulated in *cau1* while the transcript levels of *ANAC019* and *ANAC072* were similar to those in the wild-type (Fig. 1C). This result indicated that *ANAC055*, but not *ANAC019* and *ANAC072*, is suppressed by *CAU1* in drought tolerance. *ANAC019* and *ANAC072* may have redundant functions with *ANAC055*, and may be controlled by different regulators.

### *CAU1–* *ANAC055* plays a major role in drought response redundantly with *CAU1–CAS*

*CAU1* may function as a signal junction. Previous studies have shown that *CAU1* suppresses *FLC* to mediate flowering time (Pei *et al.*, 2007; Wang *et al.*, 2007; Schmitz *et al.*, 2008; Deng *et al.*, 2010), *LSM4* to mediate salt tolerance (Zhang *et al.*, 2011), bHLH to mediate iron homeostasis (Fan *et al.*, 2014), *CAS* to mediate the [Ca^2+^]_o_ signal (Fu *et al.*, 2013), *CRN* to maintain the shoot apical meristem (Yue *et al.*, 2013), *PRR7/PRR9/GI* to mediate circadian rhythms (Deng *et al.*, 2016; Li *et al.*, 2016), *PRP8* to mediate diverse developmental processes (Deng *et al.*, 2016), and *SHR* to maintain root stem cells after DNA damage (Li *et al.*, 2016). These results indicate that *CAU1* may serve as a signal junction to regulate different downstream genes in diverse biological and developmental processes.

Our results show that *CAU1* acts in response to drought, and regulates two genetically downstream pathways to modulate tolerance to drought. One downstream pathway is *CAS*-[Ca^2+^]_cyt_, which might involve ROS or IP3 signals (Fu *et al.*, 2013). The other downstream pathway is *ANAC055*–*P5CS1*, which leads to proline accumulation (Fig. 7). *CAU1*–*ANAC055* may function redundantly with *CAU1*–*CAS* in drought tolerance. The expression of *CAS* was unaffected in the *anac055* mutant (Fig. 6A), and the expressions of *ANAC055* and *P5CS1* were unaffected in the *cas-1* mutant (Fig. 6B). The stomatal closure and water loss phenotypes in *cau1* were partially restored in the *cas-1* mutant (Fu *et al.*, 2013) and restored in the *anac055* mutant (Fig. 3). These results indicate that *ANAC055* and *CAS* are independent genetically downstream genes of *CAU1*, and that *CAU1*–*ANAC055* plays a major role in response to drought stress.

### CAU1 suppresses *ANAC055* expression via histone modification

CAU1/SKB1/AtPRMT5 is an arginine methyltransferase 5 that regulates target genes by histone methylation or pre-mRNA splicing (Pei *et al.*, 2007; Wang *et al.*, 2007; Zhang *et al.*, 2011; Fu *et al.*, 2013; Yue *et al.*, 2013; Fan *et al.*, 2014; Deng *et al.*, 2016; Li *et al.*, 2016). The results of this study show that CAU1 suppresses the expression of *ANAC055* by histone methylation. The transcript levels of *ANAC055* and *P5CS1* were significantly increased in *cau1* (Figs 1C and 4D). Under PEG treatments, *CAU1* mRNA and CAU1 protein levels significantly decreased (Fig. 1A, B), and the histone methylation level decreased in *ANAC055* chromatin (Fig. 2B, D). Alternatively, spliced transcripts of *ANAC055* and *P5CS1* were not detected in *cau1* (see Fig. S3 at Dryad).

In summary, these functional analyses reveal that CAU1 serves as an epigenetic suppressor of *ANAC055*, which regulates the expression of *P5CS1*, and hence proline accumulation in response to drought stress. The results also show that the *CAU1*–*ANAC055* pathway is redundant with the *CAU1*–*CAS* pathway.

## Data deposition

The following figures and table are available at the Dryad Data Repository: https://doi.org/10.5061/dryad.hc4bj

Fig. S1. Isolation of T-DNA insertion lines for *anac055*.

Fig. S2. Transcript levels of *P5CR, PDH1* and *P5CDH*.

Fig. S3. Analysis of alternative splicing of *ANAC055* and *P5CS1*.

Table S1. List of primer sequences.

## Abbreviations:

ChIP-qPCR: chromatin immunoprecipitation quantitative PCR
H3K4me3: trimethylates histone H3 at lysine 4
P5CDH: P5C dehydrogenase
P5CR: P5C reductase
P5CS: Δ^1^-pyrroline-5-carboxylate synthetase
PDH: proline dehydrogenase
PLC: phospholipase C
PLD: phospholipase D
ROS: reactive oxygen species.
